# A Comparison of Children Born Preterm and Full-Term on the Autism Spectrum in a Prospective Community Sample

**DOI:** 10.3389/fneur.2020.597505

**Published:** 2020-12-03

**Authors:** Jenny Luu, Rachel Jellett, Maya Yaari, Melissa Gilbert, Josephine Barbaro

**Affiliations:** ^1^Olga Tennison Autism Research Centre, School of Psychology and Public Health, La Trobe University, Melbourne, VIC, Australia; ^2^Goshen – Community Child Health and Well-Being, Haruv Campus for Children, Jerusalem, Israel; ^3^Cooperative Research Centre for Living with Autism (Autism CRC), The University of Queensland, Indooroopilly, QLD, Australia

**Keywords:** prematurity, preterm, autism spectrum disorder, child development, social development, restricted repetitive behavior

## Abstract

**Introduction:** Previous research suggests children diagnosed with autism spectrum disorder (ASD or “autism”) born extremely and very preterm face substantially delayed development than their peers born full-term. Further, children born preterm are proposed to show a unique behavioral phenotype, which may overlap with characteristics of autism, making it difficult to disentangle their clinical presentation. To clarify the presentation of autism in children born preterm, this study examined differences in key indicators of child development (expressive language, receptive language, fine motor, and visual reception) and characteristics of autism (social affect and repetitive, restricted behaviors).

**Materials and Methods:** One fifty-eight children (136 full-term, twenty-two preterm) diagnosed with autism, aged 22–34 months, were identified prospectively using the Social Attention and Communication Surveillance tools during community-based, developmental surveillance checks in the second year of life. Those identified at “high likelihood” of an autism diagnosis were administered the Mullen Scales of Early Learning and the Autism Diagnostic Observation Schedule.

**Results:** The children born preterm and full-term did not differ significantly in their fine motor, visual reception, expressive language, or receptive language skills. No significant differences in social affect and repetitive and restrictive behavior traits were found.

**Discussion:** The findings of this study differs from previous research where children diagnosed with autism born very or extremely preterm were developmentally delayed and had greater autistic traits than their term-born peers. These null findings may relate to the large proportion of children born moderate to late preterm in this sample. This study was unique in its use of a community-based, prospectively identified sample of children diagnosed with autism at an early age. It may be that children in these groups differ from clinic- and hospital-based samples, that potential differences emerge later in development, or that within the autism spectrum, children born preterm and full-term develop similarly. It was concluded that within the current sample, at 2 years of age, children diagnosed with autism born preterm are similar to their peers born full-term. Thus, when clinicians identify characteristics of autism in children born preterm, it is important to refer the child for a diagnostic assessment for autism.

## Introduction

Two key areas of development characterize a diagnosis of autism spectrum disorder (ASD), hereafter “autism”: differences in social-communication (e.g., eye contact and interest in peers) and restricted, repetitive patterns of behavior (RRBs; e.g., fixated interests and stereotyped motor movements) (1). For children born preterm, there is a risk of early markers of autism, such as atypical eye gaze and protodeclarative pointing (2), being misattributed to the long-term effects from their preterm birth (3), as these can also be observed in children born preterm who do not go on to be diagnosed with autism (4, 5), despite the higher than expected prevalence of autism in children born preterm (6). This has the potential to delay diagnosis and appropriate support.

As survival rates following preterm birth increase with medical advances, more becomes known about the developmental outcomes of children born preterm (7). Children are considered preterm if they were born prior to or during the thirty-sixth week of gestation and full-term if they were born between the thirty-seventh and forty-second weeks of gestation (8). In Australia, 8.50% of children are born preterm (9). This is comparable to the estimated rate of 8.60% for developed regions and lower than the world-wide average estimated rate of 11.10% (10). There are several classifications for preterm birth based on gestational age: extremely preterm (<28 weeks gestation), very preterm (28–32 weeks gestation), and moderate to late preterm (32–36 weeks gestation) (11). Moderate to late preterm births account for 84.70% of preterm births across the world (12). Across the classifications for prematurity, children born preterm have a higher likelihood for developmental difficulties, such as having a neurodevelopmental disability (13) or meeting Diagnostic and Statistical Manual of Mental Disorders (DSM) criteria for any mental health disorder (6, 14). Although there is no single known cause, several factors may increase the likelihood for preterm birth, such as multiple gestation, maternal ethnicity, and maternal age (15). A gene by environment interaction process is likely in the etiology of preterm birth (3).

By comparing children born preterm to children born full-term, Johnson and Marlow (16) identified and described a “preterm phenotype” characterized by a distinct pattern of behaviors. The preterm phenotype is believed to result in higher rates of attentional, cognitive, and socio-emotional difficulties that can be evident across the lifespan (16, 17). These difficulties have been attributed to reduced intrauterine development of the nervous system and complications typically associated with preterm birth (3). Atypical early life experiences [e.g., over-stimulation from the neonatal intensive care unit (NICU) environment] associated with preterm birth may contribute to differences in early brain development (18). While those born preterm are often described as needing to “catch up” to those born full-term, these structural differences typically continue throughout childhood development into adulthood (19). This suggests that, rather than being delayed, brain development after preterm birth has its own trajectory (16, 19).

Research on development after preterm birth largely focuses on extreme and very preterm birth; however, a “dose effect” (17) can be observed across prematurity, in which likelihood for developmental concerns is inversely associated with gestational age. The effects of moderate to late preterm birth, where the impact of dose effect would be weakest, can be observed throughout childhood and adolescence. For example, at 2 years of age, children born moderate to late preterm were twice as likely to have a neuromotor or sensory impairment when compared to children born full-term (13). In a meta-analysis of seventy-four studies, children and adolescents born preterm had significantly lower full scale and performance intelligence quotients than their full-term counterparts (20). A dose effect was observed, with effect sizes ranging from medium and large for children born extremely preterm, reducing to small effects for children born moderate to late preterm (20). Interestingly, children born moderate to late preterm were not significantly different than their peers born full-term on verbal intelligence (20), indicating that some differences in development from preterm birth are less clear, or even undetectable, for children born moderate to late preterm.

The association between autism and preterm birth has been investigated to better understand the potential implications of preterm birth on social development. For children under the age of three, there is an estimated prevalence of 7.00% for autism in children born preterm (21). This is substantially higher than population estimates of autism for children in that age group with an estimated prevalence in the United States at 0.02% (22) or Sweden at 0.80% (23). It has been suggested that, as preterm brain development has its own trajectory (16, 19), autism may manifest differently in children born preterm (3, 24). Some evidence for this hypothesis can be found when examining cerebellum development in children on the spectrum born preterm (25–27). Further, many risk factors for autism are common characteristics of preterm births, such as low birth weight (28, 29), birth complications, more days in spent in hospital following birth (30–32), maternal infection (33), and being born small for gestational age (29). There is emerging evidence for a relationship between preterm birth and autism diagnoses, with prevalence rates of autism having an inverse relationship with gestation age (6). This suggests the importance of examining autism in preterm populations across the different categories for preterm birth to understand the dose effect across development. The relationship between preterm birth and autism is further complicated by the hypothesis that children born preterm with subtle autism traits are misdiagnosed, as their atypical behavior is attributed to their preterm birth rather than a neurodevelopmental condition (3, 16). Common markers for autism include gaze aversion and inconsistent or lack of social smiling; both behaviors that can be observed in infants born preterm with and without autism (34–36). Furthermore, children born preterm are more likely to have visual and/or hearing impairments (37, 38), which may result in atypical eye contact or response to name and subsequently a false positive screen for autism (39). Thus, developmental difficulties related to preterm birth further entangle the presentation of development after preterm birth with autism.

Attempts have been made to identify early markers for autism specific to preterm populations with mixed results. One study found that 9 month old (corrected age) infants born preterm who showed typical eye contact and gaze behavior were more likely to be screened as “high likelihood” for autism using screening measures (40). Corrected aged is used for children born preterm to account for their expected development had they been born full-term and is calculated subtracting the number of weeks born preterm from the child's chronological age. This finding was surprising as atypical eye contact and gaze are normally reliable markers for autism in young children (41–43). However, another study using a matched sample of NICU infants on the autism spectrum and not on the autism spectrum found infants later diagnosed with autism displayed expected patterns of atypical eye contact as early as 1 month (corrected age) when compared to infants not on the autism spectrum (44). Given the inconsistent results, it is unclear if children on the spectrum born preterm show the same key early markers as children on the spectrum born full-term and how these markers relate to children not on the spectrum born preterm.

Currently, there are no clear indicators of autism specific to children born preterm. However, retrospective (45) and prospective (42) studies have reliably identified early markers of autism in infants and toddlers in the general population. Specifically, atypical social-communication behavior can accurately differentiate between children on the spectrum and not on the spectrum in the first years of life (41, 43), whereas the presence of RRBs does not clearly distinguish autism from other developmental differences, such as global developmental delay (41, 43). As a result, developmental surveillance with an emphasis on early social-communication behaviors, rather than RRBs, has been shown to be effective in identifying children at an increased likelihood for autism (2). This is beneficial, as a reliable diagnosis can be made by 2 years of age (46) with early identification and diagnosis having positive impact on future development as compared to later diagnosis (47–49).

Barbaro et al. (2, 42, 50–53) used social-communication markers to develop a universal tool to monitor infants and toddlers for autism, the Social Attention and Communication Surveillance (SACS) tool. The SACS tool and its revised version (SACS-R) (53), were developed for use in community settings to prospectively identify children between 12 and 30 months of age who display a pattern of atypical behaviors indicating a higher likelihood of autism. Development is monitored at 6-month intervals based on age-expected behaviors. A strength of the SACS tool is in its positive predictive value (the probability that children with a positive screening test truly have autism) of 81.00–83.00% (42, 53) between 12 and 24 months of age, which is higher than other commonly used autism screening tools for young children, such as the Modified Checklist for Autism in Toddlers (M-CHAT) (54) with a positive predictive value of 6.00% (55) and the Ages and Stages Questionnaire with a positive predictive value between 26.70 and 30.30% (56). While there have been previous studies in preterm children using the M-CHAT, discussed in further detail below, thus far no studies have targeted infants and toddlers on the spectrum born preterm who were identified using the SACS.

In addition, few studies have explicitly examined differences between preterm and full-term groups with an autism diagnosis. Two studies considering the impact of preterm birth on social-communication presentation found greater autism behaviors for children born very or moderate to late preterm (57, 58). Five year old children on the spectrum were identified to have a specific weakness in social reciprocity compared to their peers born full-term on the Autism Diagnostic Interview-Revised (ADI-R) (59). However, no differences were found for the same domain on the clinical observation measure of the Autism Diagnostic Observation Schedule (ADOS) (57, 60). An additional study using the Revised Behavior Summarized Evaluation Scale (61), another observational measure, found no significant differences for social-communication behaviors in young children on the spectrum born moderate to late preterm (62). Differences in the other key criteria for autism diagnosis, RRBs, has not been well researched between preterm and full-term groups. Using the ADOS and ADI-R, no differences were found in RRB presentation for 5 year old children on the spectrum born preterm and full-term on either measure (57). Another study focusing on children born moderate to late preterm at 5 years of age used the Repetitive and Restrictive Behavior Scale (63), which includes four subscales: sensorimotor stereotypies, reaction to chance, restricted behaviors, and modulation insufficiency. No significant differences were found on any of the subscales between the preterm and full-term groups (62). While there is consistency in the findings of these two studies, presentation of RRBs at earlier stages of development for children on the spectrum born preterm has yet to be examined.

As with research on non-autistic children born preterm, previous research on children on the spectrum born preterm indicates that these children are more likely to have delayed development than their term-born peers. Several studies have compared preterm and full-term groups that were identified at “high likelihood” for autism using autism screening measures. These studies found that children born preterm had lower overall development scores across cognitive, language, and motor developmental profiles than their term-born peers, with medium to large effect sizes (64, 65). When considering older children and adolescents on the spectrum, those born preterm are more likely to be non-verbal compared to those born full-term indicating that differences in cognitive development can be identified by 3 years of age (62, 66). Identifying potential differences in developmental profiles would be useful in identifying additional needs that children born preterm may have as a group, though no known studies thus far have examined this.

Clinical uncertainty pertaining to diagnosis of autism in preterm populations could impact the care provided to these children and, consequently, their development. Concerningly, a meta-analysis found that the median age for diagnosis in children born preterm was 5.7 years of age (21) while the average age for diagnosis in Australia (67) and the United States (68) are both 4 years of age. Potentially, being born preterm may delay assessment, diagnosis, and the opportunity to access early supports that can improve developmental outcomes.

To date, no known studies have compared children on the spectrum born preterm and full-term within a prospectively identified, community-based sample. This study aimed to identify differences in developmental profiles and autistic trait presentation in children on the spectrum born preterm and full-term aged 22–34 months who were identified from a community-based sample. It was hypothesized that children on the spectrum born preterm would have lower developmental quotients than children on the spectrum born full-term for receptive language, expressive language, fine motor, and visual reception. Further, when comparing children on the spectrum, it was hypothesized that those born preterm would have greater autistic presentation than those born full-term for social communication on clinical observation measures. Due to the limited number of studies that have investigated differences in RRB presentation between children on the spectrum born preterm and full-term, no hypotheses were made for this young sample.

## Method

### Participants

Participants were drawn from two existing prospective, community-based, studies: the SACS (42) and SACS-R (53). Between the two studies, 35,732 children from Victoria, Australia were monitored between 11 and 30 months of age using the SACS tools, resulting in 357 children identified at “high likelihood” for autism.

Of these, 218 children underwent diagnostic assessment at 2 years of age. After excluding children whose gestational age or birth weight was unknown (*n* = 3 preterm, *n* = 55 full-term), one twin born preterm (to retain independence of observations), one child born preterm with an incomplete assessment, the final sample included twenty-two children born preterm and 136 children born full-term with an autism diagnosis, aged 22–34 months at the time of assessment. Of the children born preterm, one (4.50%) was born very preterm, with twenty-one (95.50%) born moderate to late preterm; no child was born extremely preterm. Approximately half of the children born preterm (*n* = 11) were born in the thirty-sixth week of gestation (see Table 1).

**Table 1:** Number of children born in each week of gestation within the preterm and full-term groups.

**Classification**	**Number of weeks gestation**	**Number of children**
Very preterm	31	1
Moderate to late preterm	32	2
	33	2
	34	2
	35	3
	36	11
Full-term	37	11
	38	30
	39	35
	40	35
	41	19

### Measures

The SACS (42) and SACS-R (53) are universal, community-based screening tools for monitoring children between 11 and 30 months of age to identify those with a “high likelihood” of autism. Trained raters mark whether a child displays typical or atypical behavior against several items, the number and content of which differs at each age as the items are based on developmental expectations. Each assessment has five “key items” for autism and a number of “non-key” items, as identified in Barbaro and Dissanayake (2). Children who are rated as having atypical behavior on at least three of the “key items” for their age group are deemed at “high likelihood” for autism. The SACS and SACS-R tools both have overall positive predictive values of 81.00–83.00%, negative predictive value of 99.00%, sensitivity of 82.00–84.00%, and specificity of 99.00–99.50% for identifying children on the spectrum between 12 and 24 months of age, and an inter-rater reliability of 0.90 (42, 53).

Parents/caregivers completed a demographic questionnaire, reporting on characteristics of their family, education level, culture, occupation, income, and language/s spoken at home. The demographic questionnaire in the SACS-R study had additional questions on whether siblings or other family members had a diagnosis of autism. Information about the child's birth was recorded via the demographics questionnaire, notes provided by maternal and child health (MCH) nurses, documentation from families during their visit, or in photocopies from the “My Health, Learning and Development Record” birth record books provided to families in Victoria when their child is born. Birth and development information is recorded in these books by hospital and MCH nurses.

The Mullen Scales of Early Learning (MSEL) (69) was used to examine developmental profiles for children. This task-based measure for children aged between 3 and 68 months of age includes subscales of fine motor, visual reception, receptive language, and expressive language skills. In the current study, the MSEL subscales had excellent internal reliability (α = 0.75–0.91). Further, the MSEL has been validated for use with young children with an autism diagnosis with excellent construct validity between 0.84 and 0.92 (70). Per procedure for the MSEL (69), corrected age was used when the child's chronological age was under 24 months and chronological age used thereafter. Developmental quotients for subscales were calculated by dividing scale age equivalents by the child's chronological or corrected age and multiplying by one hundred.

The ADOS is a semi-structured, standardized, play-based assessment with modules administered based on the child's age and language development. Module 1 of the ADOS-Generic (ADOS-G) (71) was used in the SACS study, as appropriate for the children's age and language development. In the SACS-R study, children aged between 12 and 30 months completed the ADOS-Toddler Module (ADOS-T) (72) or the ADOS-2 Modules 1–2 (60) were administered as appropriate for their language level. Items are coded between zero and two, with higher scores indicating greater autism traits. A Cochrane review of the ADOS-G, ADOS-T and ADOS-2 modules found a summary sensitivity of 0.94 and specificity of 0.80 in preschool aged children (73).

To allow for comparisons between the different ADOS versions and modules, algorithms for calibrated severity scores (CSSs) were created for social affect, RRBs, and overall severity, using the method proposed by Hus et al. (74), Gotham et al. (75), and Esler et al. (76). Higher CSSs indicate greater autism traits, ranging from zero to ten. However, the possible range of scores for RRB CSSs is zero or between five and ten, skipping numbers one to four. These algorithms had internal reliability coefficients ranging from acceptable (ADOS-G; α = 0.68) to excellent (ADOS-T and ADOS-2 all modules; α = 0.73–0.91) within this study. The process to calculate CSSs is frequently used in autism research to allow comparisons across editions and modules of the ADOS (57, 77).

### Procedure

Ethics approval from the La Trobe University Human Ethics Committee was obtained for the SACS (Project 06-94) and SACS-R (UHEC13-001) prior to data collection. An application for this secondary data analysis was approved prior to commencement (HEC-19209).

Across both studies, MCH centers from nineteen local government areas (LGAs) across Melbourne, Victoria took part, with five LGAs taking part in both studies. The MCH service provides caregivers with a schedule of free consultations with MCH nurses for ten “key ages and stages” of development in the first 6 years of life (78). Nurses from MCH attended a half-day workshop on identifying early behavioral signs of autism (42, 53). Children attending routine MCH appointments in Victoria, Australia were subsequently screened using the SACS or SACS-R tools at all scheduled 12-, 18-, and 24-month “key ages and stages” appointments between September 2006 to September 2008 for the SACS study (42) and June 2013 to June 2018 for the SACS-R study (53).

All children who were identified at “high likelihood” for an autism diagnosis were invited to attend developmental assessments at the University's Child Development Unit at six-monthly intervals to track their development over time. Parents provided informed consent for their child's assessment, MCH records, and photocopies made from their “My Health, Learning and Development Record” books to be used for the SACS/SACS-R research and future studies. At the developmental assessment for children at 2-years of age, parent/caregiver and child measures were completed in tandem, with one clinician administering the ADOS and MSEL to the child while another clinician interviewed the parent(s) or caregiver(s). An assessment report was provided for the family after each appointment.

#### Preliminary Analyses

Prior to analysis, assumptions were tested. The level of measurement assumption was met as dependent variables were continuous. As children were tested independently and one child from a pair of twins was removed from analysis, the assumption of independence of observations was met. Normality was assessed using visual inspection of histograms, skewness z-scores with magnitude >0.5, and Kolmogorov-Smirnov and Shapiro-Wilks Tests of Normality. The assumption of normality was violated for all MSEL developmental quotients (except receptive language) and ADOS RRB CSSs due to negative skew. When cell sizes are ≥20, multivariate analyses of variances (MANOVAs) are robust against violations to normality, so no transformations were made (79). Two multivariate outliers were detected from the full-term group and were not removed as they accounted for <5.00% of the participants in that group (80).

Assumptions required for MANOVAs were tested to examine the MSEL development quotients and ADOS CSSs. Box's Test was not significant, indicating the assumption of variance-covariance matrices was met. Levene's Test was not significant for any of the MANOVAs, indicating that the assumption of equality of variances was met.

#### Statistical Analyses

Pearson's correlations were used to identify relationships between gestational age, birth weight, and the dependent measures. To identify differences in birth characteristics between the preterm group and full-term group, *t*-tests and Fisher's Exact Test were used. Fisher's Exact Test was used instead of chi-squared tests when the assumption of minimum cell frequency was not met, specifically, ≥10 cases per cell for 2x2 tables and ≥5 cases per cell for 2x3 tables. Given Fisher's Exact Test with tables larger than 2x2 is not available within the Statistical Package for Social Sciences (SPSS) (81), Fisher's Exact Test (2-tailed) with Freeman-Halton extension for 2x3 tables was calculated using VassarStats (82).

To examine group differences between the preterm and full-term groups, a MANOVA was used to examine the MSEL developmental quotients and another MANOVA for social affect and RRB ADOS CSSs. To determine whether a difference in age between the preterm and full-term groups affected the main results, an MANCOVA was perform on MSEL developmental quotients and ADOS CSSs.

## Results

Correlations were calculated to determine the strength of the relationships between gestational age, birth weight, and the outcome measures (see Table 2). While the correlation between birth weight and gestational age was significant, neither were significantly correlated with any of the outcome measures. Correlations between the MSEL developmental quotients were significant, with weak to large positive correlations (83). The ADOS social affect and RRB CSSs were significantly and positively correlated with moderate strength (see Table 2).

**Table 2:** Correlations between gestational age, birth weight, Mullen Scales of Early Learning developmental quotients, and Autism Diagnostic Observation Schedule calibrated severity scores.

**Variable**	**BW**	**VR DQ**	**FM DQ**	**RL DQ**	**EL DQ**	**SA CSS**	**RRB CSS**
GA	0.68^**^	0.00	0.07	−0.03	−0.04	0.08	0.05
BW	–	0.01	0	0	−0.01	0.06	0.01
VR DQ		–	0.70^**^	0.66^**^	0.64^**^	−0.36^**^	−0.24^**^
FM DQ			–	0.46^**^	0.43^**^	−0.33^**^	−0.19^*^
RL DQ				–	0.77^**^	−0.41^**^	−0.24^**^
EL DQ					–	−0.28^**^	−0.16^*^
SA CSS						–	0.23^*^

*GA, gestational age; BW, birth weight; DQ, Mullen Scales of Early Learning developmental quotient; CSS, Autism Diagnostic Observation Schedule calibrated severity score; VR, visual reception; FM, fine motor; RL, receptive language; EL, expressive language; SA, social affect; RRB, repetitive and restricted behavior*.

* *p < 0.05 (2-tailed)*.

** *p < 0.01 (2-tailed)*.

Children in the preterm group had significantly lower gestational age and birth weight and were significantly more likely to have been born small for gestational age and have a complication at birth than children in the full-term group (see Table 3). Children in the preterm group were significantly older than children in the full-term group in chronological age. After controlling for chronological age, the results of main analyses remain the same (see Supplementary Table 1).

**Table 3:** Differences in birth characteristics and demographics of preterm and full-term groups.

	**Preterm**		**Full-term**					
**Continuous variables**	***n***	***M* (*SD*)**	***n***	***M* (*SD*)**	***t***	***df***	***p***	***d***
Age (months)	22	27.32 (2.72)	136	26.01 (2.53)	2.24	156	0.03	0.50
Gestational age (weeks)	22	35.17 (1.42)	136	39.37 (1.23)	14.61	156	<0.001	3.18
Birth weight (g)	22	2,395.91 (609.44)	136	3,485.27 (493.00)	9.30	156	<0.001	1.97
**Categorical variables**	***n***	**%**	***n***	**%**	**Fisher's Exact Test** ***p***			
Sex					0.768			
Males	19	86.4	110	80.9				
Female	3	13.6	26	19.1				
Birth Weight <2,500 g					<0.001			
No	14	63.6	136	100.0				
Yes	8	36.4	0	0.0				
Size for gestational age					0.167			
Small	6	27.3	17	12.5				
Average	13	59.1	103	75.7				
Large	3	13.6	16	11.8				
Complications at birth					<0.001			
Yes	18	81.8	42	31.6				
No	4	18.2	91	68.4				

*Equal variances assumed for t-tests. n, number of participants; M (SD), Mean (Standard Deviation)*.

No significant differences were found between the preterm and full-term groups on any of the MSEL development quotients for visual reception, fine motor, receptive language, and expressive language between children on the spectrum born preterm and full-term. Further, there were no significant differences in behavior presentation using the ADOS CSSs for social affect and RRBs (see Table 4).

**Table 4:** MANOVA results for differences between preterm and full-term groups on Mullen Scales of Early Learning developmental quotients and Autism Diagnostic Observation Schedule calibrated severity scores.

	**Preterm**		**Full-term**								
	***n***	***M***	***SD***	***N***	***M***	***SD***	***F***	***df_**1**_, df_**2**_***	***p***	**Λ**	**$\eta_{p}^{2}$**
MSEL DQ	22			136			0.74	4, 153	0.556	0.98	0.02
VR		73.61	12.12		78.27	17.99					
FM		80.90	12.20		86.00	16.37					
RL		57.51	22.93		58.66	27.71					
EL		62.92	25.73		62.91	23.00					
ADOS CSS	22			136			0.17	2, 155	0.85	1.00	0.002
SA		6.05	1.96		6.32	2.22					
RRB		7.00	1.80		6.99	2.18					

*n, number of participants; M, mean; SD, standard deviation; F, F-statistic; df , degrees of freedom; Λ, Wilk's Lambda; $\eta_{p}^{2}$, partial eta square; MSEL DQ, Mullen Scales of Early Learning developmental quotient; VR, visual reception; FM, fine motor; RL, receptive language; EL, expressive language; ADOS CSS, Autism Diagnostic Observation Schedule calibrated severity score; SA, social affect; RRB, restricted, repetitive patterns of behavior*.

## Discussion

Early years presentation of autism was examined in children born preterm and full-term who were prospectively identified at “high likelihood” for autism from the community. The hypothesis that children on the spectrum born preterm would have more delayed development than children on the spectrum born full-term was not supported as no significant differences were identified in visual reception, fine motor, receptive language, and expressive language developmental quotients. Further, the hypothesis that children on the spectrum born preterm would have greater social-communication presentation than children on the spectrum born full-term was not supported, with no significant group differences found.

The non-significant differences across key indicators of child development between children on the spectrum born preterm and full-term were not consistent with previous literature on older children and adolescents between 3 and 18 years of age, where delayed development was observed for those born preterm with a diagnosis of autism as compared to their peers without an autism diagnosis (62, 66). Specifically, previous literature identified verbal development as being delayed and was identified in children as young as 3 years of age (62, 66). In the current study using a sample of 2-year-old children, these differences were not identified. Similar results were found for children born preterm who were identified at “high likelihood” for an autism diagnosis using screening tools (28, 65). Further, previous findings using typically developing samples have suggested that children born preterm have substantially delayed development when compared to children born full-term (20, 84).

The inconsistency of findings regarding child development after preterm birth in the current study with previous literature may be attributed to the young age of the children in this sample (22–34 months) as compared to previous studies that had included children between 3 and 18 years of age (62, 65, 66). As previous research has not yet included children within toddler age with a diagnosis of autism, it is possible that differences in development may not become apparent until the child reaches an older age. While differences were found for young children who had screened at “high likelihood” without a diagnosis of autism in previous studies (28, 65), comparing them to children with a diagnosis of autism may be problematic due to the high rates of false positives when using autism screening tools in preterm groups (56, 85). Extrapolating development of children who have screened at “high likelihood” for autism to children with a diagnosis of autism may be misleading due to the other potential explanations for a child born preterm being identified at “high likelihood” without a full developmental assessment, as was conducted in the current study.

The finding that children on the spectrum born preterm and full-term did not differ on social communication behavior presentation was not consistent with previous studies using parent report measures, where children on the spectrum born preterm were shown to have greater social-communication behavior presentation (57, 58). It is possible that more subtle differences in social-communication behaviors could be unpacked using a measure with subscales within the domains or an item-by-item analysis. Another explanation for the findings of the current study on social-communication for populations of children on the spectrum born preterm and full-term being inconsistent with previous literature may be the young sample. As with developmental profiles, it is possible that differences in behavioral presentation do not emerge until children are at an older age. While Movsas and Paneth (58) included children as young as 4 years of age in their study, the mean age of their participants was 10 years (58)—much older than the current sample's mean age of 26.20 months. Another study found differences between children on the spectrum born preterm and full-term at 5 years of age; however, only children who were born very preterm but not at a low birth weight (<1,500 grams) were excluded from this study (57). The non-significant finding of the current study is consistent with other literature using children born moderate to late preterm (57, 62). When examining samples of 5-year-old children on the spectrum born very (61) and moderate to late preterm (66), no significant differences were found on social-communication behavior presentation. This is not surprising, given 95.50% of the preterm group of the current study were born moderate to late preterm.

This study also examined potential differences in RRB presentation between children on the spectrum born preterm and full-term. The current study builds upon emerging evidence that no differences exist in this domain. Previously, no differences were found for 5 year old children on the spectrum born preterm and full-term on parent report (57) and clinical observation measures (57, 62). The sample in the current study included children who were younger than those used in these previous studies, where the youngest participants were aged 3 years in Brayette et al. (62) and 5 years in Chen et al. (57). Therefore, the findings that children on the spectrum born preterm and full-term do not differ on RRB presentation extend to earlier in development with the current sample.

Instability in patterns of autism screening have been observed in children born preterm between 8 and 18 months of age (86). In that study, half of the children born preterm who were identified at “high likelihood” for autism at 18 months of age using the M-CHAT had not been previously identified at 8 or 12 months of age (86). Further, several children suddenly no longer screened positive at eighteen, despite previously screening positive at 8 and/or 12 months of age (86). When the children were 3 years old, only one child born preterm and no children born full-term was diagnosed with autism (65). These findings may point to some children born preterm having a “sudden onset” of behaviors while others have a sudden decrease at an older age (86). It is possible that instability of RRB presentation could explain the inconsistent findings in the literature at different age groups. The diagnostic inclusion for the current study was based on the child's most recent diagnosis (had they attended subsequent follow up appointments) rather than the diagnosis received at their first appointment. Furthermore, diagnoses were based on gold standard developmental assessments and clinical judgement, rather than the presence or absence of behaviors at the age of diagnosis. Thus, while the trajectory for autistic traits seems to be difficult to predict using screening measures in preterm population, the continued developmental surveillance was advantageous in ensuring their diagnoses were accurate.

Previous studies have largely recruited from clinics and/or university hospitals, where samples typically consist of families who have concerns about their child's development (87). As parents are less likely to be aware of the subtle differences in development that may indicate that a child is at “high likelihood” of an autism diagnosis, children who attend clinics following parental concerns may have more challenging autism traits or other developmental concerns than those identified by trained clinicians. Further, when seeking advice on these subtle differences, clinicians who are unaware of the relationship between preterm birth and autism may assure parents that many of these behaviors, such as atypical eye contact (34) or toe-walking (88), are behaviors common for children born preterm without autism. Thus, autism is not considered as a potential explanation for behavior and the child is not referred to a full developmental assessment. It would not be until a child is showing a pattern of behavior which more clearly indicates autism as an explanation for behavior, that a full developmental assessment is considered. In contrast, the current study was a community-based sample where all children within the community were monitored for autism, rather than only those whose parents have concerns. This difference in sampling could account for the inconsistency between the findings of previous literature and the current study, due to the comparison of children with potentially more subtle developmental differences in both preterm and full-term groups.

Another potential explanation for the inconsistent findings across the literature may be the dose effect in the preterm phenotype, where children with lower gestational ages face more developmental difficulties (17). Previous studies (62) and the current study, which have involved children born preterm with higher gestational ages, have not detected differences in behavioral presentation; similar findings can be observed across preterm phenotype literature (3, 20). Alternatively, it may be that a subset of children born moderate to late preterm are susceptible to developmental difficulties, rather than all children born between moderate to late preterm (89). Further, Sansavini et al. (90) note that many children born extremely preterm, where occurrence of developmental difficulties would be greatest and easiest to detect due to the dose effect, are found to have development within the normal distribution for development (90). As many children born preterm have developmental scores within the range of children born full-term, differences become difficult to detect. The findings of the current study further suggest that even within children who have been diagnosed with autism, their general development is similar to children with an autism diagnosis born full-term—expanding the literature on similarities in development for children born preterm and full-term.

The preterm phenotype suggests that autism in preterm populations may have inherently different etiology than autism in full-term populations (3, 16). Children born preterm were identified at “high likelihood” and diagnosed using criteria based on full-term groups. Potentially, current diagnostic criteria may not accurately represent autism in preterm groups or difficulties might emerge later in life than in full-term groups (86). As a result, the prevalence of autism in preterm populations may be under- or over-estimated. Thus, participants in this study might reflect those with patterns of autistic traits that reflect “typical” autism for full-term populations, which may not accurately represent “typical” autism in preterm populations. While this is a limitation to the study, it is also a limitation to this area of research. Until differences in the presentation of autism in children born preterm and full-term are identified (or ruled out), using the diagnostic criteria based on full-term populations is unavoidable.

This study had several strengths in its unique contributions to an area of research in the preterm phenotype. Firstly, it was the first study to investigate developmental differences and behavioral presentation in children on the spectrum born preterm and full-term, aged 22–34 months. Development of children on the spectrum born preterm has yet to be examined in children at this age, with most studies focusing on children with a diagnosis aged 5 years or older. In younger samples, the children tend to have been identified at “high likelihood” for autism without a confirmed diagnosis, which becomes problematic due to low predictive value and instability of the screening tools used in those studies (55, 56, 86). The use of a young sample is well aligned with the current focus for autism research of early identification and support in autism (91). Secondly, the use of the SACS tools to prospectively identify children on the spectrum in the community may have allowed for more subtle presentations of autism to be detected compared to studies using other screening measures, where these children may have missed. Third, this is one of few studies using a community-based sample of children born preterm and full-term. Use of samples of children born extremely and very preterm from single hospitals are common in this research area, limiting the generalizability of their findings from children born moderate and late preterm, who comprise the majority the preterm population; thus, the use of community-based samples increases the ecological validity of the findings. Finally, as prior research has primarily focused on extremely and very preterm populations, the inclusion of the moderate to late preterm population helps fill the gap for an underserved group of children in the premature phenotype research area.

Although there are notable strengths of this study, it is not without limitations. Firstly, although rates of children born preterm identified with the SACS tools were consistent with population rates of preterm birth, a low number of children born very preterm and no children born extremely preterm were involved in the study. This did not allow for a detailed examination of the dose effect of the preterm phenotype on the outcome measures, as lesser developmental differences from moderate to late preterm birth may obscure larger differences from extreme and very preterm birth. Secondly, while the age range of the current sample was small relative to other studies, very subtle differences in development within this age group may not have been captured. However, measures that account for age and developmental norms were chosen to counteract this, resulting in children being compared based on their expected level of development. Third, children in the preterm group in the current sample were significantly older than children in the full-term group in chronological age. However, when the data were analyzed with age as a covariate, the results remained the same indicating that the difference in age did not significantly affect the results. Lastly, as children who did not have known gestational ages were excluded from the study, fewer preterm children were excluded from the study (*n* = 3) than children born full-term (*n* = 55). This discrepancy likely occurred as more care is taken to record gestational age and birth weight for children born preterm than children born full-term. Gestational age and birth weight have a much more relevant impact on development for children born preterm and the accuracy of these figures becomes more important than for a child born full-term. However, one demographic difference (paternal education) was found between the children who were excluded due to missing gestational age or birth weight when compared with children who were included in the current study (see Supplementary Table 2). As no other demographic differences were identified, and there was incomplete data on paternal education, this indicated that those included and excluded were largely similar to each other.

In future, researchers may wish to further explore the differences in RRB presentation and preterm groups using other measures that further break down RRBs into subscales for more detailed analysis. As this study did not examine gross motor performance, it is possible that children on the spectrum born preterm were unable to perform gross motor-based RRBs due to other developmental difficulties, which may have been a confounding factor in the lack of differences between the preterm and full-term groups on the RRB measure. A more detailed examination into other predictors of autism, such as birth weight, and their effect on development and behavioral presentation, may be useful in unpacking inconsistent findings on preterm autism literature. Additionally, no detailed data were available regarding participants experiencing neonatal complications, which could also account for future developmental difficulties. Further, an examination into behaviors and characteristics of children born preterm, with and without autism, would give further insight into the boundary between the preterm phenotype and autism in preterm populations. Lastly, future research using longitudinal study designs could examine the trajectories of behavioral presentation within preterm populations to determine whether, and at which age, differences become apparent.

Previous literature paints a picture of children born preterm having many additional needs due to developmental difficulties and delays. This picture may lead clinicians to expect and look for more challenging characteristics of autism when assessing a child born preterm, overlooking those presenting with more mild developmental differences and behavioral presentation. Using a community-based sample of children identified prospectively, it was found that children on the spectrum born moderate to late preterm did not differ in development and behavioral presentation from their peers born full-term, when assessed at the age of 2 years. As current autism research has largely focused on identifying children at younger ages and providing support as early as possible, the findings of the current study suggest that clinicians should consider autism as a potential explanation for behaviors that are often presumed to be due to preterm birth, particularly for children who were born moderate to late preterm. As these results indicate that the autism phenotype is similar for moderate to late preterm and full-term children, clinicians should not change their clinical approach of diagnosis and treatment for a child presenting with the characteristics of autism simply due to their preterm birth. While further research is required to replicate and extend these findings, it is possible that many 2-year-old children on the spectrum born moderate to late preterm whom clinicians meet in the community will have similar needs to their term-born peers. Still, individual differences in development should not be overlooked, particularly for children born preterm who are more likely to face additional developmental difficulties.

As autism research moves to improving early identification, these findings have practical implications for clinicians who may overlook autism as an explanation for behavior due to expectations for greater developmental differences in children born preterm. Further, these findings may provide reassurance to families, who may have concerns for their child's support needs and outcomes after an autism diagnosis.

## Data Availability Statement

The data analyzed in this study are subject to the following licenses/restrictions: the original contributions presented in the study are included in the article/supplementary material. The data required to reproduce these findings cannot be shared at this time as the data also forms part of an ongoing study. Requests to access these datasets should be directed to Josephine Barbaro, j.barbaro@latrobe.edu.au.

## Ethics Statement

The studies involving human participants were reviewed and approved by La Trobe University Human Ethics Committee. Written informed consent to participate in this study was provided by the participants' legal guardian/next of kin.

## Author Contributions

JL conducted the analyses, contributed to the interpretation of the results, and drafted the initial manuscript. RJ and JB provided supervision to JL, assisted with study design, data analysis and interpretation, and reviewed drafts. MY assisted with the study design and interpretation of results. MG assisted with data analysis and interpretation of results. All authors contributed to the article and approved the submitted version.

## Conflict of Interest

The authors declare that the research was conducted in the absence of any commercial or financial relationships that could be construed as a potential conflict of interest.
