# Supplementary material for: A Comparison of Children Born Preterm and Full-Term on the Autism Spectrum in a Prospective Community Sample
Source: Front Neurol. 2020 Dec 3;11:597505. doi: 10.3389/fneur.2020.597505 (PMC7744721; doi:10.3389/fneur.2020.597505)
Supplement: Supplementary file 1 [file Table_1.docx]

Supplementary Material

# Supplementary Figures and Tables

## Supplementary table 1.

*One-way MANCOVA Results for Differences Between Preterm and Full-Term Groups on Mullen Scales of Early Learning Developmental Quotients and Autism Diagnostic Observation Schedule Calibrated Severity Scores, controlling for chronological age at assessment.*

|  | Preterm | | |  | Full-term | | |  |  |  |  |  |
| --- | --- | --- | --- | --- | --- | --- | --- | --- | --- | --- | --- | --- |
|  | *n* | *M* | *SD* |  | *n* | *M* | *SD* | *F* | *df_1_, df_2_* | *p* | Λ | η_p_^2^ |
| MSEL DQ  VR  FM  RL  EL | 22 | 73.61  80.90  57.51  62.92 | 12.12  12.20  22.93  25.73 |  | 136 | 78.27  86.00  58.66  62.91 | 17.99  16.37  27.71  23.00 | 0.34 | 4, 152 | .853 | 0.99 | 0.01 |
| ADOS CSS  SA  RRB | 22 | 6.05  7.00 | 1.96  1.80 |  | 136 | 6.32  6.99 | 2.22  2.18 | 0.23 | 2, 154 | .80 | 1.00 | 0.003 |

*Note*. *n* = number of participants. *M* = mean. *SD* = standard deviation. *F* = *F*-statistic. *df* = degrees of freedom. Λ = Wilk’s Lambda. η_p_^2^  = partial eta square. MSEL DQ = Mullen Scales of Early Learning developmental quotient. VR = visual reception. FM = fine motor. RL = receptive language. EL = expressive language. ADOS CSS = Autism Diagnostic Observation Schedule calibrated severity score. SA = social affect. RRB = restricted, repetitive patterns of behavior.

## Supplementary table 2

*Demographic Comparison of Children Born Preterm and Full-Term Excluded Based on Missing Birthweight or Gestational Age and Children Included in Current Study.*

|  | Excluded | |  | Included | |  | Fisher’s |  |  |  |
| --- | --- | --- | --- | --- | --- | --- | --- | --- | --- | --- |
|  | *n* | % |  | *n* | % |  | *p* | ꭓ^2^ | *df* | *p* |
| Sex  Males  Female | 44  14 | 75.9  24.1 |  | 130  30 | 81.3  23.7 |  | .445 |  |  |  |
| Birth Complications  No  Yes | 6  95 | 5.9  94.1 |  | 6  62 | 9.1  90.9 |  | .547 |  |  |  |
| Labour Type  Spontaneous  Induced  No labour | 6  5  0 | 54.5  45.5  0.0 |  | 75  34  9 | 63.6  28.8  7.6 |  |  | 1.9 | 2 | .389 |
| ICU/SCN after birth  No  Yes | 7  1 | 87.5  12.5 |  | 107  21 | 83.6  16.6 |  |  | 0.1 | 1 | .771 |
| Maternal culture  European  Asian/Middle Eastern  Other | 30  16  6 | 57.7  30.7  11.5 |  | 91  32  14 | 66.4  23.4  10.2 |  |  | 1.3 | 2 | .515 |
| Paternal culture  European  Asian/Middle Eastern  Other | 23  13  10 | 50.0  28.3  21.7 |  | 81  31  20 | 61.4  23.5  15.1 |  |  | 1.9 | 2 | .378 |
| Second Language spoken at home  No  Yes | 2  57 | 3.4  96.6 |  | 0  7 | 0.0  100.0 |  | 1.00 |  |  |  |
| Maternal Education  Secondary  Trade/TAFE/Tertiary | 12  46 | 28.7  79.3 |  | 95  111 | 28.8  71.2 |  |  | 1.4 | 1 | .236 |
| Paternal Education  Secondary  Trade/TAFE/Tertiary | 10  36 | 21.8  79.2 |  | 56  87 | 40.6  59.4 |  |  | 4.6 | 1 | .031* |

Note: *** = *p* < .05

Fisher’s = Fisher’s Exact Test, ICU = Intensive Care Unit, SCN = Special Care Nursery, TAFE = Technical and Further Education
